# Population normative data for OxCAP-MH capability scores

**DOI:** 10.1007/s10198-024-01696-w

**Published:** 2024-05-24

**Authors:** Péter György Balázs, Agata Łaszewska, Judit Simon, Valentin Brodszky

**Affiliations:** 1 Department of Health Policy, Corvinus University of Budapest, Budapest, Hungary; 2 Department of Health Economics, Medical University of Vienna, Vienna, Austria; 3 Department of Psychiatry, University of Oxford, Oxford, UK

**Keywords:** Population norm, OxCAP-MH, Capability measurement, Well-being assessment, Mental health status

## Abstract

**Aim:**

The study aims to establish the first set of normative data for OxCAP-MH capability instrument and to examine its association with sociodemographic and anxiety/depression severity variables.

**Methods:**

A large-sample cross-sectional online survey was conducted among the Hungarian adult general population in 2021. OxCAP-MH standardized mean scores were compared across age, sex, education level, residence, employment, and marital status. Linear regression analysis was employed to determine the impact of sociodemographic and anxiety/depression severity on the OxCAP-MH score.

**Results:**

In total, N = 2000 individuals completed the survey. The sample mean age was 47.1, with female majority (53.4%). Most respondents had completed primary education (51%), were active on labour market (52.4%), lived in larger cities (70.0%), and were married/in relationship (61.1%). Nearly half of the participants reported experiencing depression (48.5%), anxiety (44.3%), and 38.6% reported having both. The mean OxCAP-MH score for the total sample was 67.2 (SD = 14.4), the highest in the non-depressed (74.4) and non-anxious (73.6) subgroups, the lowest among those with extremely severe depression (45.0) and severe anxiety (47.7). Regression results indicated that older individuals (by β = 0.1), males (β = 2.3), those with secondary or higher education (β = 2.7 and 4.5) and students (β = 6.8) had significantly (p<0.01) higher mental capabilities. Respondents with mild, moderate, severe, or extremely severe depression (β =  -6.6, -9.6, -13.8, -18.3) and those with mild, moderate, or severe anxiety (β =  -4.1, -7.7, -10.3) had lower capability scores.

**Conclusion:**

The OxCAP-MH instrument effectively differentiated capabilities across sociodemographic groups and highlighting the impact of depression and anxiety severity on general population’s mental capability.

**Supplementary Information:**

The online version contains supplementary material available at 10.1007/s10198-024-01696-w.

## Introduction

Most country-specific guidelines recommend the use of quality-adjusted life year (QALY) measure to express health gain in health economic evaluations [1]. A QALY integrates quantity of life (as life expectancy) and quality of life (expressed through health state utility) into a single measure. Evaluation guidelines predominantly advise to consider the preferences of the general population when weighting the utility, as they are the payers of the health care system [2].

Increasing prevalence of chronic mental diseases and the mental health repercussions of the recent events such as the COVID-19 pandemic and other social stressors (e.g. inflation related economic concerns, migration, war in Ukraine) place an increasing burden on the health and social care systems. Health economic evaluations often consider QALY, that rests on health-related quality of life (HRQoL) measurements, as golden standard, since it can capture quality weighted health outcome. However, recent experiences of challenging events have underscored the need for broader measures that go beyond HRQoL and cover wider concept of well-being [3, 4]. There is a growing need in health intervention assessments to extend the scope of health outcome analysis beyond physiological and psychological health and mitigate the inherent narrow nature of QALY. Apart from HRQoL, that covers strictly health-related life domains, broader well-being aspects are becoming more relevant in recent years [5]. Certain public health interventions (such as long term care, community mental health services, programs for drug recovery and rehabilitation) are hardly to be captured by QALY in light of that, health state utility measures and HRQoL instruments are limited to health-related domains only, which can lead to a potential underestimation of the outcomes resulting from such interventions [6]. More researchers argue that healthcare demands should be evaluated from a broader social welfare perspective [7]. Health state utility measurements rely on rational choice theory and gauge respondents health quality [8], whereas capability instruments are rooted in Amartya Sen’s capability approach, aiming to capture the overall well-being of individuals [9]. Capability instruments provide a comprehensive assessment of individual’s emotional, social, mental and physical functioning and capture changes in overall well-being, that is relevant in preventive and long term care interventions, where traditional HRQoL instruments might be myopic. Mapping a broader range of intervention outcomes, that allows to improve the capabilities of disadvantaged populations, promotes social justice.

In accordance with international recommendations, capability measures hold practical relevance in social care and economic evaluations [10–12]. Preference and non-preference weighted forms of capability outcome measures are being extensively used in economic evaluations [13]. A growing number of capability questionnaires and empirical studies also reflect the increasing interest towards capability assessment. Over the past 15 years, fourteen capability instruments have been developed, some of which widely expanded internationally [7].

The Oxford CAPabilities-Mental Health questionnaire (OxCAP-MH) was developed to be used in mental health-related (economic) evaluations to measure levels of relevant capabilities [14]. The theory of the capability approach emphasizes the individual’s ability and freedom to acquire objectives they value. Much like the frequently used ICEpop CAPability (ICECAP-A/O) capability measure for Adults/Older and Adult Social Care Outcomes Toolkit (ASCOT) for social care outcomes, the OxCAP-MH was developed in the UK [15, 16]. It has since five further language validations: German [17], Hungarian [18], Luganda [19], Juba Arabic, and Chinese. Previous studies have reported sound psychometric properties and consistent factor structure of the OxCAP-MH instrument [17, 19–22]. Besides cognitive debriefings and psychometric assessments, former OxCAP-MH studies involved disease specific (people living with HIV/AIDS and depression, schizophrenia) samples [18, 19, 23] but no population norms have been established so far. Consequently, a large-sample study, based on the general population is essential to interpret the OxCAP-MH scores across different mental health severity groups along with impacts of sociodemographic characteristics.

This study endeavours to establish the first OxCAP-MH population norms based on a large representative general population sample in Hungary. The complementary aim was to assess the association between capability scores, sociodemographic characteristics and mental health status.

## Materials and methods

### Survey

A large, self-administrated, online survey was conducted among the Hungarian general non-institutionalised population in August 2021. Respondents were recruited from a panel database of a survey company. Population composition ‘soft’ quotas were set to obtain a representative sample in terms of age, gender, level of education and residence. Respondents, who were ≥ 18 years old participated voluntarily and received points that could be redeemed for financial rewards. The study was approved by the Research Ethics Committee of the Corvinus University of Budapest (no. KRH/166/2021). The questionnaire consisted of validated instruments related to health status, well-being, informal care, healthcare resource use, and sociodemographic questions.

### Instruments

The *OxCAP-MH* consists of 16 items, each scored on a 1–5 Likert scale (total raw score range of 16–80), where five refers to the highest level of capabilities and one refers to the lowest level of capabilities. Items 2,4,5,6,9–16 are reverse coded. The change in scoring pattern ought to avoid pattern answering, though slows interpretation. Standardised capability scores are expressed on an easy to read 0 to 100 scale, where 100 represents the highest level of capabilities. The standardized capability score is calculated as: 100 * (OxCAP-MH item total score-minimum score of 16)/(max-min score).

The scale was originally designed to measure capabilities of people suffering from mental health disorders. The measurement tool covers several well-being domains (including health and non-health dimension) – such as limitations in daily activities, social activities, recreation, influence on decisions, freedom of expression – that determine capabilities of individuals [14, 24]. The culturally and linguistically validated Hungarian language version of the OxCAP-MH was used [18].

*Patient Health Questionnaire 9-item* (PHQ-9) was developed to measure self-experienced severity of depression [25]. The nine questions request information from the past two weeks and are based on the Diagnostic and Statistical Manual of Mental Disorders criteria for major depressive disorder. Items are rated on a 4-point Likert scale, where 0 means “not at all” 3 denotes “nearly every day”. Item sum score ranges between 0 and 27. Originally, four severity categories were established: no (0–4), mild (5–9), moderate (10–14), severe (15–19), extremely severe depression (20–27). Many studies investigated the cut-off score to signify severity of depression, commonly setting the sensitivity of the instrument as: non-depressed (score below 10) or depressed (score of 10 or more) [26–29].

*Generalized Anxiety Disorder 7-item* scale (GAD-7) is a self-reported measurement tool that assesses anxiety symptom severity on a 0–3 point Likert scale describing frequency of experienced symptoms (not at all - nearly every day) during the last two weeks. Item responses are summed resulting in a score ranging between 0 and 21. Individual results are interpreted as no (0–4), mild (5–9), moderate (10–14), severe (15+) anxiety [30, 31].

### Analysis

Sample characteristics were presented as weighted descriptive statistics for the total sample (*N* = 2000). National micro census data were presented to discern sample and population composition [32]. The sample-data were weighted based on representativeness quotas for age, sex, education and residency to extrapolate result for the whole Hungarian general population. The population normative data of OxCAP-MH standardized score were presented according to age groups (18–24; 25–34; 35–44; 45–54; 55–64; 65≤), by sex (males and females), education level (primary, secondary, tertiary), residence (Budapest, town, village), employment status (fill-time employed/entrepreneur, part-time employed, unemployed, student, retired, disability pensioner, other/homemaker) and marital status (single, married/in permanent relationship, divorced/widowed), and by mental-health status (PHQ-9: no 0–4, mild 5–9, moderate 10–14, severe 15–19, extremely severe depression 20–27 and GAD-7: no 0–4, mild 5–9, moderate 10–14, severe anxiety 15–21) subgroups (Table 1).

**Table 1:** Observed variables and subgroups for OxCAP-MH normative data

Characteristics	Categories
*Age group*	18–24; 25–34; 35–44; 45–54; 55–64; 65≤ (in regression input as scale)
*Sex*	male; female (reference coded)
*Level of education*	primary (reference coded); secondary; tertiary
*Residence type*	Budapest; town; village (less than 10,000 inhabitants, reference coded)
*Employment status*	full-time employed/entrepreneur; part-time employed; unemployed (reference coded); student; retired; inactive/disability pensioner; other such as homemaker/caregiver
*Marital status*	married/in permanent relationship, single (reference coded); divorced/widowed
*PHQ-9 severity level (0–27)*	no (reference coded); mild; moderate; severe; extremely severe depression
*GAD-7 severity level (0–21)*	no (reference coded); mild; moderate; severe anxiety

The arithmetic mean and standard deviations (SD) of the OxCAP-MH standardized scores are reported. Item response distributions were observed. All analyses were performed in STATA (16.0).

Means (standard deviation, SD) of OxCAP-MH were compared with nonparametric t-tests analysing the significant differences (*p* < 0.05) between sociodemographic and mental health subgroups. To investigate the determinants of capabilities, an ordinary least square (OLS) multivariate regression was used, robust for survey sample data and heteroscedasticity (survey set weight = sample-weight, variance-covariance matrix = linearized):

$$\begin{array}{l}OxCAPM{H_i} = {\beta _0} + {\beta _i}Age + {\beta _i}Sex + {\beta _i}Educ + {\beta _i}Resid\\+ {\beta _i}Employ + {\beta _i}Maritalst + {\beta _i}Dep + {\gamma _i}Anx + {\varepsilon _i}\end{array}$$

## Results

### Sample characteristics

The sample consists of *N* = 2000 complete responses (response rate: 78%) from the Hungarian adult general population, with mean age of 47.1 (SD = 16.6) and majority being female (53.4%). Most respondents had primary education (51.0%), worked as full-time employees or as entrepreneurs (46.5%), lived in towns (51.9%), and were married or lived in permanent relationship (61.1%). The depressed (PHQ-9 score < 10) and non-depressed (PHQ-9 score ≥ 10) subsamples have roughly the same characteristics with respect to age, sex, education level, residency, employment status and marital status.

Among the total sample 48.5% of respondents had depression, 44.3% anxiety and 38.6% had both according to PHQ-9 and GAD-7. Severe/extreme level of depression was experienced by 12.2% and 6.6% had severe anxiety (Table 2).

**Table 2:** Study sample characteristics

Variable	Subgroups	Total sample		Non-depressed group (PHQ-9 < 10)		Depressed group (PHQ-9 score ≥ 10)		Population composition	
		***n***	**%**	***n***	**%**	***n***	**%**	***n***	**%**
Sex	male	933	46.6	756	49.5	177	37.5	6,677,542	47.7
	female	1067	53.4	772	50.5	295	62.5	5,126,295	52.3
Age (group)	18–24	212	10.6	140	9.2	72	15.2	1,100,392	11.2
	25–34	338	16.9	240	15.7	99	20.8	1,230,306	12.6
	35–44	376	18.8	272	17.8	103	21.9	1,576,249	16.1
	45–54	309	15.5	232	15.2	77	16.3	1,295,469	13.2
	55–64	352	17.6	280	18.3	72	15.3	1,358,200	13.9
	65≤	412	20.6	363	23.7	49	10.4	1,821,284	18.6
Level of education	primary	1020	51.0	741	48.5	279	58.1	4,141,685	41.7
	secondary	627	31.3	483	31.6	144	30.5	4,307,921	43.3
	tertiary	354	17.7	304	19.9	49	10.4	1,488,022	15.0
Residence	Budapest	362	18.1	282	18.5	80	17.0	1,706,851	17.6
	Town	1038	51.9	808	52.9	231	48.8	5,083,374	52.5
	Countryside	599	30.0	438	28.7	161	34.2	2,898785	29.9
Employment status	full-time employed /entrepreneur	929	46.5	721	47.2	208	44.0	4,586,300	47.3
	part-time employed	119	5.9	84	5.5	35	7.4		
	unemployed	95	4.8	62	4.0	34	7.1	173,800	1.8
	student	58	2.9	42	2.7	16	3.4	279,100	2.9
	retired	509	25.4	439	28.8	69	14.6	2,465,500	25.5
	other (homemaker)	202	10.1	133	8.7	69	14.6	776,200	8.0
	disability pensioner/inactive	89	4.4	47	3.1	42	8.9	1,408,100	14.5
Marital status	single	417	23.6	324	21.2	147	31.2	4,298,781	43.9
	married/in relationship	1222	61.1	969	63.4	253	53.5	3,686,969	37.6
	divorced/widowed	307	15.4	235	15.4	72	15.3	1,818,087	18.6
PHQ-9 depression category	no	1031	51.5	1031	67.5	-		n/a	
	mild	497	24.8	497	32.5	-			
	moderate	230	11.5	-		230	48.7		
	severe	149	7.5	-		149	31.6		
	extremely severe	93	4.7	-		93	19.8		
GAD-7 anxiety category	no	1115	55.7	1088	71.2	27	5.7	n/a	
	mild	516	25.8	372	24.4	144	30.4		
	moderate	237	11.8	63	4.2	173	36.7		
	severe	133	6.6	4	0.3	129	27.2		

*Note: n/a refers to no accessible information

### OxCAP-MH item response distribution

**Fig. 1:**
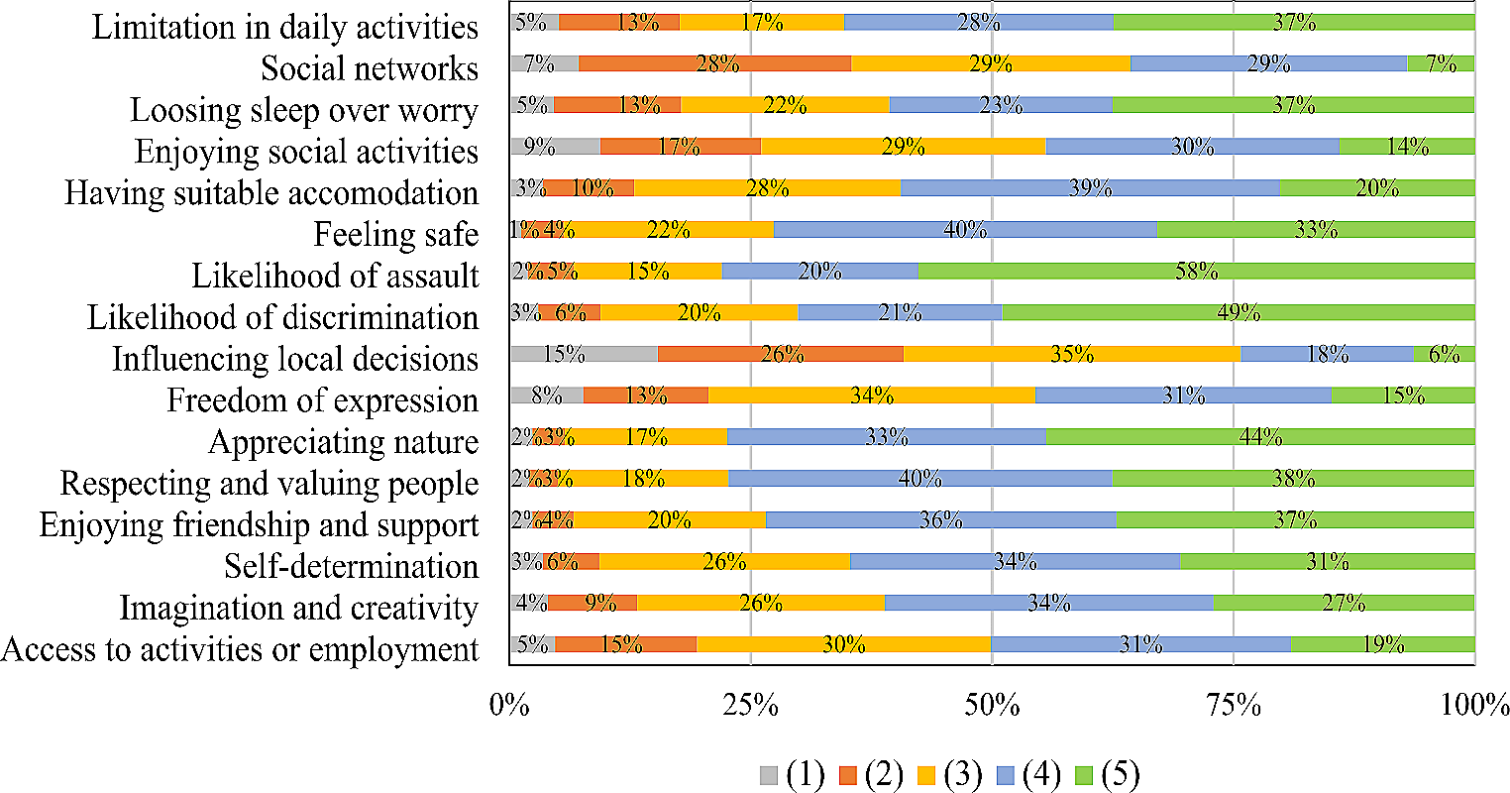
OxCAP-MH item response distribution among total sample

The item responses distributions present, that most people were somehow limited in influencing local decisions (Item-9: 94%), maintaining social networks (Item-2: 93%) and enjoying social activities (Item-4: 86%), only 6%, 7% and 14% gave full capability responses (Fig. 1). The worst capability results were in influencing local decisions, 15% of sample population indicated total inability and 26% limited ability. The respondents had the best capabilities in perceptions about the future, most were not afraid of future assault (Item-7: 58%), not worried over future discrimination (Item-8: 49%) and a great proportion was able to appreciate nature (Item-11: 44%).

### Population normative data for OxCAP-MH

The total sample mean (SD) OxCAP-MH score was 67.2 (14.4), where males had slightly, but significantly (*p* < 0.01) higher 67.7 (15.2) capability scores then females 66.9 (14.4). People with higher education aged 65≤, residents of Budapest/larger cities, students/retired, divorced/widowed had better capabilities (mean scores: 72.6, 71.3, 70.4, 68.3, 67.9), respectively. Respondents without depression and without anxiety had overall the highest mean capability scores (74.4 and 73.6), while the worst capabilities were among extremely depressed and severely anxious (mean: 45.0 and 47.7). OxCAP-MH score significantly differed in all sociodemographic and mental health status subgroups (*p* < 0.01) (Table 3).

**Table 3:** OxCAP-MH population normative data as mean (SD) scores

Variables	Subgroups	total	18–24	25–34	35–44	45–54	55–64	65≤
Total	all respondents	67.2 (14.8)	63.6 (15.6)	65.4 (13.9)	65.0 (14.8)	68.5 (14.6)	67.6 (15.1)	71.3 (13.7)
Sex	male	67.7 (15.2)	61.9 (16.2)	65.4 (14.7)	64.6 (16.4)	70.0 (14.8)	68.9 (13.9)	72.0 (13.4)
	female	66.9 (14.4)	65.0 (14.9)	65.4 (12.9)	65.4 (12.9)	67.2 (14.3)	66.5 (16.1)	70.6 (14.1)
Level of education	primary	64.4 (15.0)	56.7 (15.0)	63.0 (14.1)	61.9 (14.4)	66.9 (14.4)	64.2 (15.5)	69.1 (14.8)
	secondary	68.8 (14.1)	67.9 (14.4)	65.6 (13.7)	67.1 (15.4)	72.2 (13.6)	70.4 (14.2)	71.2 (12.7)
	tertiary	72.6 (12.8)	70.3 (13.0)	69.0 (13.3)	71.0 (12.5)	73.2 (15.7)	75.7 (10.5)	75.0 (12.2)
Residence	Budapest	67.9 (14.4)	61.0 (11.5)	64.5 (14.2)	68.2 (14.6)	68.8 (12.2)	72.2 (12.5)	71.1 (16.0)
	Town	67.9 (14.6)	65.8 (15.3)	67.1 (14.0)	65.1 (14.9)	68.9 (15.5)	67.5 (15.2)	71.7 (12.3)
	Countryside	65.6 (15.2)	61.0 (18.1)	62.7 (13.2)	63.0 (14.7)	67.7 (14.1)	65.8 (15.7)	70.6 (14.6)
Employment status	full-time employed/entrepreneur	68.2 (14.0)	64.7 (15.3)	66.5 (14.1)	67.1 (13.9)	71.0 (13.9)	69.7 (12.7)	78.0 (11.3)
	part-time employed	62.2 (14.7)	61.4 (12.9)	59.0 (13.6)	58.8 (20.0)	64.5 (12.2)	62.6 (10.9)	72.5 (12.1)
	unemployed	62.1 (14.5)	60.0 (15.0)	59.6 (14.3)	67.4 (12.1)	63.0 (16.5)	57.8 (13.6)	84.4 (-)**
	student	70.4 (14.3)	71.7 (13.6)	55.3 (20.8)*	-	-	64.1 (0.0)*	-
	retired	70.4 (14.6)	37.5 (0.0)*	53.2 (2.9)*	43.5 (17.7)	62.8 (18.3)	71.9 (14.8)	71.2 (13.8)
	other (e.g. homemaker)	62.9 (14.4)	55.1 (14.8)	67.0 (12.3)	60.1 (12.9)	68.5 (11.9)	64.8 (17.9)	67.8 (15.3)
	disability pensioner/inactive	59.1 (15.6)	57.7 (10.7)	56.2 (7.9)	59.7 (11.7)	61.8 (15.6)	57.9 (19.1)	62.0 (8.9)
Marital status	single	64.2 (15.6)	64.4 (16.0)	61.6 (14.4)	63.2 (15.9)	66.1 (16.8)	68.1 (15.1)	68.3 (14.1)
	married/in relationship	68.1 (14.7)	63.6 (15.4)	67.2 (13.3)	66.1 (14.4)	70.2 (13.6)	67.6 (15.9)	71.4 (14.6)
	divorced/widowed	68.3 (13.1)	54.6 (7.6)	64.5 (16.9)	62.3 (11.8)	65.4 (14.4)	67.5 (12.5)	71.6 (12.0)
PHQ-9 (depression)	no	74.4 (12.6)	72.1 (14.9)	73.0 (12.9)	71.9 (13.4)	75.9 (12.4)	74.5 (12.5)	76.7 (10.6)
	mild	64.9 (11.4)	64.2 (13.2)	63.7 (10.7)	64.4 (11.9)	64.9 (11.7)	64.7 (10.2)	67.0 (11.2)
	moderate	58.6 (10.3)	57.6 (10.9)	57.7 (9.9)	59.1 (8.8)	60.6 (9.4)	57.8 (13.9)	59.2 (8.6)
	severe	52.6 (11.4)	54.0 (8.1)	54.1 (8.9)	50.0 (12.2)	54.7 (12.7)	52.0 (13.8)	52.1 (11.5)
	extremely severe	45.0 (12.0)	45.9 (13.2)	46.8 (8.7)	44.7 (9.2)	50.0 (13.2)	41.6 (15.2)	37.6 (10.7)
GAD-7 (anxiety)	no	73.6 (12.4)	72.5 (13.3)	72.4 (13.0)	71.0 (13.1)	75.1 (12.6)	73.6 (12.0)	75.4 (11.4)
	mild	63.8 (12.1)	62.5 (13.1)	63.6 (11.5)	62.7 (12.7)	64.4 (11.9)	64.5 (12.9)	65.8 (10.4)
	moderate	55.6 (12.0)	54.2 (11.6)	56.3 (8.3)	54.9 (15.2)	55.6 (11.5)	55.0 (11.8)	58.6 (13.2)
	severe	47.7 (12.1)	46.5 (14.8)	49.3 (9.8)	50.5 (10.3)	52.1 (12.3)	41.1 (13.4)	43.5 (13.0)

Note: *denotes groups where responses are < 5, **refers to *n* = 1 respondent

### Factors associated with OxCAP-MH capability well-being

The OLS multivariate linear regression results show that the eight observed factors explain 40.3% of the variation in the dependent variable, the overall model was significant (*p* < 0.001). Four sociodemographic and two mental health status variables were significantly associated with capability well-being. Respondents in older age (β = 0.09), males (β = 2.27), with secondary and higher education (β = 2.65 and β = 4.53), and students (β = 6.75) had significantly higher OxCAP-MH capability scores (*p* < 0.01) than younger individuals, females, people with primary education and unemployed. Compared to non-depressed and non-anxious, respondents with mild/moderate/severe/extremely severe depression (β = -6.55, -9.86, -13.80, -18.34) and with mild/moderate/severe anxiety (β = -4.11, -7.71, -10.30) had significantly (*p* < 0.001) lower OxCAP-MH capability scores. The mild-moderate level of depression had about 1.5 times more impact than mild-moderate anxiety on capabilities, while severe level of depression had nearly double the negative effect then severe anxiety (Table 4).

**Table 4:** Regression analysis results of factors associated with OxCAP-MH capability well-being

Variable	Subgroups	β coefficient	SE	*p*-value
Age	(as years)	***0.094***	***0.032***	***0.003***
Sex	male	***2.269***	***0.639***	***< 0.001***
Education level	secondary	***2.647***	***0.670***	***< 0.001***
	tertiary	***4.530***	***0.738***	***< 0.001***
Residence	Budapest	0.634	0.872	0.468
	Town	1.162	0.688	0.091
Employment status	full-time employed/entrepreneur	2.416	1.542	0.117
	part-time employed	-1.109	2.086	0.595
	student	6.754	2.041	***< 0.001***
	retired	-0.035	1.820	0.985
	other (homemaker)	0.561	1.819	0.758
	disability pensioner	-1.705	2.204	0.439
Marital status	married/in relationship	1.162	0.853	0.173
	widowed/divorced	1.290	1.137	0.257
PHQ-9 (depression)	mild	***-6.552***	***0.857***	***< 0.001***
	moderate	***-9.862***	***1.232***	***< 0.001***
	severe	***-13.797***	***1.666***	***< 0.001***
	extremely severe	***-18.342***	***2.452***	***< 0.001***
GAD-7 (anxiety)	mild	***-4.109***	***0.885***	***< 0.001***
	moderate	***-7.714***	***1.330***	***< 0.001***
	severe	***-10.297***	***2.187***	***< 0.001***
*Regression indices*	*Constant*	*66.662*	*2.331*	*< 0.001*
	*N observation*	2000		
	*R²*	40.3%		
	*significance*	*p* < 0.001		

*Note: in all items (1) refers to lowest, (5) refers to highest capabilities

## Discussion and conclusion

The primary objective of the study was to establish the first large sample of general population normative dataset for OxCAP-MH capabilities. A total of *N* = 2000 respondents completed the online, self-administrated questionnaire in August 2021. The Hungarian general population’s OxCAP-MH mean scores (67.2) after the COVID-19 compared to the Austrian general population scores (74.1) during the first lockdown are considerably lower and rather similar to means of Austrian patients with mental health diagnoses (64.0) [3, 20]. The highest capabilities were observed in people with no depression (74.4), while the lowest in extremely severe depression (45.0). Consistently with the total sample, males and females had the best OxCAP-MH capabilities in non-depressed (74.1 and 74.6) and non-anxious (72.8 and 74.5) subgroups., Considering that the lowest scores were observed in extremely depressed subgroups (46.8 for males and 43.9 for females) and the high proportion of severely depressed in the total sample (12.2%), the capability results of the Hungarian general population falling beneath the Austrian general population and being closer to the Austrian and United Kingdom mental health disorder patients’ outcomes is not surprising [23].

Influence on local decisions (Item-9) seem to be an extreme among respondents’ capability domains, that is possibly considered as an exogeneous factor, being rather politically determined and independent of Hungarian respondents’ personal capabilities. Alternatively, the panel data may provide a snapshot of post-covid circumstances, while the data collection took place in 2021 summer right after the end of the third COVID-19 wave 2021 spring. Until then several governmental crisis decisions were made centrally (e.g. on lockdown/restrictions, public and private service operations, opening hours), which could lower the confidence in yielding impact upon the local-level decision-making. As a further consequence of lockdowns, the enjoyment in social activities and extent of social networks also yielded the lowest capabilities (Item-2 and Item-4), that may partly explain the significantly decreased well-being of younger populations.

Regarding the sociodemographic characteristics, similar associative patters were noted in Hungary, when comparing capability and previous EQ-5D population norm, that is female gender and lower education level decreases capability and HRQoL [33]. However, a previous ICECAP-A/O population norm study in Hungary found no significant associations in relation of capabilities and age, gender, education level, and self-experienced health state our study revealed that older respondents, males, secondary and tertiary educated have better OxCAP-MH capability mean scores by 0.1%, 2.3%, 2.7% and 4.5% respectively. While those with worse self-reported depression and anxiety severity had significantly lower (-6.6%, -9.9%, -13.8%, -18.3% for depression and  -4.1%, -7.7%, -10.3% for anxiety) OxCAP-MH capability scores.

OxCAP-MH measure was (1) able to clearly differentiate between subgroups according to sociodemographic characteristics and mental state severity, also (2) exhibited a strong association with the severity of mental health states.

An increasing number of well-being instruments are applied in care-service and outcome measure in public health fields: Measure of Achieved Capabilities in Homeless services (MACHS) [34], Central Human Capabilities (referred to as OCAP-18) [6], or assessment of self-reported capabilities [35]. These instruments are also tailored to specific target populations (e.g. ICECAP has version for adults and older people [12], ASCOT for caregivers and people in need [36], OxCAP-MH is originally designed for people with mental health impairments). Therefore, evaluations shall consider choosing the most accurate measurement tool available for decision-support analysis.

The study limitations enfold (1) the data quality issues of panel database, (2) study representativeness generated by weighting the non-representative large sample of institutionalized population (excluding prisoners, people require nursing, ethnical minorities, etc.), (3) reliance on non-clinically verified, self-reported mental health status measures.

Conclusively, this study presents the first large-sample population normative data for OxCAP-MH capability scores. Our results support that the instrument can be used in a general population setting, while still captures robust difference among different mental health state severity groups. Significantly higher capability scores were observed in older individuals, males, those with higher education, students and respondents with no depression/no anxiety.

## Electronic supplementary material

Below is the link to the electronic supplementary material.

Supplementary Material 1
