# Supplementary material for: Population normative data for OxCAP-MH capability scores
Source: Eur J Health Econ. 2024 May 24;26(2):223–31. doi: 10.1007/s10198-024-01696-w (PMC11889034; doi:10.1007/s10198-024-01696-w)
Supplement: Supplementary file 1 — Supplementary Material 1 [file 10198_2024_1696_MOESM1_ESM.docx]

*Appendix 1: Population normative data of the Hungarian general population according to OxCAP-MH capability standardized mean score and (SD):*

| **Variable/**  **subgroups** | | **Total** | | | | | | | **Female** | | | | | | | **Male** | | | | | | |
| --- | --- | --- | --- | --- | --- | --- | --- | --- | --- | --- | --- | --- | --- | --- | --- | --- | --- | --- | --- | --- | --- | --- |
|  |  | **total** | **18-24** | **25-34** | **35-44** | **45-54** | **55-64** | **65<** | **total** | **18-24** | **25-34** | **35-44** | **45-54** | **55-64** | **65<** | **total** | **18-24** | **25-34** | **35-44** | **45-54** | **55-64** | **65<** |
| **Education level** | primary | 64.4 (15.0) | 56.7 (15.0) | 63.0 (14.1) | 61.9 (14.4) | 66.9 (14.4) | 64.2 (15.5) | 69.1 (14.8) | 64.0 (14.5) | 58.6 (14.3) | 62.8 (13.6) | 63.6 (10.8) | 65.0 (13.9) | 61.8 (16.6) | 69.3 (14.5) | 64.9 (15.8) | 54.4 (15.6) | 63.5 (15.4) | 60.3 (17.0) | 69.2 (14.7) | 66.7 (14.0) | 68.9 (14.2) |
|  | secondary | 68.8 (14.1) | 67.9 (14.4) | 65.6 (13.7) | 67.1 (15.4) | 72.2 (13.6) | 70.4 (14.2) | 71.2 (12.7) | 69.0 (14.0) | 69.1 (14.0) | 65.4 (13.0) | 65.0 (16.3) | 73.8 (13.3) | 71.1 (13.9) | 71.3 (13.0) | 68.5 (14.3) | 66.8 (14.8) | 65.6 (14.8) | 68.7 (14.6) | 70.8 (13.9) | 69.4 (14.6) | 71.1 (12.4) |
|  | tertiary | 72.6 (12.8) | 70.3 (13.0) | 69.0 (13.3) | 71.0 (12.5) | 73.2 (15.7) | 75.7 (10.5) | 75.0 (12.2) | 71.8 (12.6) | 70.0 (13.7) | 69.8 (13.2) | 71.3 (11.3) | 74.3 (14.2) | 75.8 (10.8) | 72.4 (13.0) | 73.4 (13.0) | 72.7 (7.1)* | 67.8 (13.5) | 70.7 (13.8) | 72.5 (16.9) | 75.5 (10.3) | 76.3 (11.7) |
| **Residence** | Budapest | 67.9 (14.4) | 61.0 (11.5) | 64.5 (14.2) | 68.2 (14.6) | 68.8 (12.2) | 72.2 (12.5) | 71.1 (16.0) | 67.9 (13.3) | 63.1 (11.9) | 64.8 (13.8) | 70.2 (11.0) | 68.6 (12.1) | 68.1 (12.7) | 71.5 (15.4) | 68.0 (15.3) | 58.6 (10.7) | 64.1 (14.9) | 66.8 (16.6) | 69.1 (12.7) | 76.5 (11.0) | 70.7 (16.6) |
|  | Town | 67.9 (14.6) | 65.8 (15.3) | 67.1 (14.0) | 65.1 (14.9) | 68.9 (15.5) | 67.5 (15.2) | 71.7 (12.3) | 67.6 (14.3) | 67.5 (15.3) | 66.2 (13.5) | 64.9 (13.7) | 66.1 (15.2) | 69.2 (16.3) | 71.0 (12.0) | 68.2 (14.9) | 63.5 (15.2) | 68.9 (14.9) | 65.2 (15.7) | 71.8 (15.3) | 66.1 (14.1) | 72.4 (12.7) |
|  | Countryside | 65.6 (15.2) | 61.0 (18.1) | 62.7 (13.2) | 63.0 (14.7) | 67.7 (14.1) | 65.8 (15.7) | 70.6 (14.6) | 65.1 (14.8) | 60.5 (15.4) | 64.2 (13.6) | 64.0 (12.5) | 68.2 (13.7) | 62.8 (16.3) | 69.3 (16.7) | 66.2 (15.6) | 61.5 (20.4) | 59.5 (11.8) | 61.5 (17.6) | 67.1 (14.7) | 70.7 (13.3) | 72.0 (12.0) |
| **Employment status** | full-time employed/  entrepreneur | 68.2 (14.0) | 64.7 (15.3) | 66.5 (14.1) | 67.1 (13.9) | 71.0 (13.9) | 69.7 (12.7) | 78.0 (11.3) | 67.5 (13.7) | 69.1 (14.5) | 65.8 (14.2) | 67.9 (13.1) | 69.9 (13.9) | 65.1 (12.3) | 74.8 (12.1)* | 68.7 (14.3) | 60.8 (15.2) | 67.4 (14.1) | 66.6 (14.5) | 71.9 (13.9) | 73.1 (12.0) | 79.3 (11.3) |
|  | part-time  employed | 62.2 (14.7) | 61.4 (12.9) | 59.0 (13.6) | 58.8 (20.0) | 64.5 (12.2) | 62.6 (10.9) | 72.5 (12.1) | 62.2 (11.9) | 57.3 (8.2) | 58.1 (14.9) | 63.2 (12.4) | 61.9 (10.8) | 64.4 (13.1) | 69.9 (10.8)* | 62.2 (17.1) | 64.7 (15.5) | 60.1 (13.0) | 55.5 (24.1) | 67.6 (13.4) | 60.8 (8.4) | 74.9 (14.5)* |
|  | unemployed | 62.1 (14.5) | 60.0 (15.0) | 59.6 (14.3) | 67.4 (12.1) | 63.0 (16.5) | 57.8 (13.6) | 84.4  (-)** | 61.4 (13.7) | 53.3 (14.5) | 60.2 (11.6) | 65.5 (12.2) | 61.5 (16.3) | 61.2 (16.5) | - | 63.2 (15.9) | 66.3 (13.4) | 57.6 (21.7) | 74.4 (9.9) | 66.3 (17.9) | 55.6 (11.8) | 84.4  (-)** |
|  | student | 70.4 (14.3) | 71.7 (13.6) | 55.3 (20.8)* | - | - | 64.1 (0.0)* | - | 71.8 (13.5) | 72.2 (13.3) | 64.1 (21.0)* | - | - | - | - | 68.8 (15.4) | 70.9 (14.3) | 47.6 (25.2)* | - | - | 64.1 (0.0)* | - |
|  | retired | 70.4 (14.6) | 37.5 (0.0)* | 53.2 (2.9)* | 43.5 (17.7) | 62.8 (18.3) | 71.9 (14.8) | 71.2 (13.8) | 71.0 (14.6) | 37.5 (0.0)* | - | - | 58.5 (18.5) | 74.3 (13.3) | 70.7 (14.2) | 69.8 (15.0) | - | 53.2 (2.9)* | 43.5 (17.7) | 65.4 (18.4) | 66.9 (16.5) | 71.7 (13.4) |
|  | other (e.g. homemaker) | 62.9 (14.4) | 55.1 (14.8) | 67.0 (12.3) | 60.1 (12.9) | 68.5 (11.9) | 64.8 (17.9) | 67.8 (15.3) | 63.7 (13.6) | 58.2 (12.4) | 67.1 (12.4) | 60.9 (12.2) | 68.5 (12.1) | 62.8 (21.1) | 61.1 (14.6)* | 57.3 (17.7) | 47.1 (17.8) | 61.7  (-)** | 53.7 (18.0) | 71.9  (-)** | 69.2 (7.7) | 77.1 (17.5)* |
|  | disab.pens./  inactive | 59.1 (15.6) | 57.7 (10.7) | 56.2 (7.9) | 59.7 (11.7) | 61.8 (15.6) | 57.9 (19.1) | 62.0 (8.9) | 58.6 (17.0) | 65.0 (12.9)* | 58.9 (10.3)* | 61.0 (4.8)* | 64.6 (15.3) | 51.9 (19.2) | 71.9 (0.0)** | 59.7 (14.1) | 54.8 (9.5) | 54.2 (6.0) | 58.6 (16.7)* | 53.5 (14.7) | 65.4 (16.6) | 58.7 (6.3)* |
| **Marital status** | single | 64.2 (15.6) | 64.4 (16.0) | 61.6 (14.4) | 63.2 (15.9) | 66.1 (16.8) | 68.1 (15.1) | 68.3 (14.1) | 63.1 (14.7) | 63.7 (15.9) | 59.9 (13.9) | 65.1 (13.0) | 62.9 (14.4) | 65.4 (17.8) | 66.9 (13.3) | 65.1 (16.3) | 65.0 (16.3) | 63.9 (15.0) | 62.5 (16.8) | 68.3 (18.1) | 69.9 (13.1) | 71.1 (16.0) |
|  | married/in relationship | 68.1 (14.7) | 63.6 (15.4) | 67.2 (13.3) | 66.1 (14.4) | 70.2 (13.6) | 67.6 (15.9) | 71.4 (14.6) | 67.4 (14.5) | 66.3 (14.3) | 67.5 (12.7) | 65.7 (13.2) | 69.5 (13.5) | 66.1 (17.3) | 69.9 (16.1) | 68.9 (14.8) | 58.9 (16.4) | 66.5 (14.5) | 66.8 (15.9) | 71.1 (13.7) | 69.0 (14.3) | 72.3 (13.7) |
|  | divorced/  widowed | 68.3 (13.1) | 54.6 (7.6) | 64.5 (16.9) | 62.3 (11.8) | 65.4 (14.4) | 67.5 (12.5) | 71.6 (12.0) | 68.9 (13.1) | 63.6 (7.2)* | 69.5 (20.9)* | 62.7 (8.3) | 64.2 (15.4) | 67.8 (12.3) | 72.0 (12.2) | 67.1 (13.0) | 51.1 (4.2) | 60.6 (15.2)* | 61.6 (16.7) | 68.1 (11.8) | 67.0 (13.2) | 70.7 (11.6) |
| **PHQ-9 category** | no | 74.4 (12.6) | 72.1 (14.9) | 73.0 (12.9) | 71.9 (13.4) | 75.9 (12.4) | 74.5 (12.5) | 76.7 (10.6) | 74.6 (12.2) | 70.8 (16.6) | 72.8 (12.6) | 73.9 (11.4) | 74.9 (12.0) | 76.1 (11.8) | 76.8 (10.3) | 74.1 (12.9) | 73.6 (12.6) | 73.3 (13.4) | 70.4 (14.5) | 76.8 (12.7) | 73.2 (12.9) | 76.6 (10.9) |
|  | mild | 64.9 (11.4) | 64.2 (13.2) | 63.7 (10.7) | 64.4 (11.9) | 64.9 (11.7) | 64.7 (10.2) | 67.0 (11.2) | 66.2 (10.7) | 68.8 (10.4) | 64.4 (10.7) | 64.4 (8.3) | 66.4 (12.0) | 64.3 (10.9) | 68.8 (10.5) | 63.1 (12.1) | 58.9 (14.2) | 61.9 (10.9) | 64.4 (15.2) | 62.9 (11.0) | 65.1 (9.3) | 63.7 (11.3) |
|  | moderate | 58.6 (10.3) | 57.6 (10.9) | 57.7 (9.9) | 59.1 (8.8) | 60.6 (9.4) | 57.8 (13.9) | 59.2 (8.6) | 59.2 (10.9) | 59.3 (11.5) | 58.3 (11.0) | 59.8 (10.1) | 59.6 (7.8) | 58.7 (14.5) | 60.2 (9.1) | 57.8 (9.3) | 55.8 (10.2) | 56.6 (8.0) | 58.5 (7.3) | 62.1 (11.5) | 55.1 (12.0) | 56.9 (7.2) |
|  | severe | 52.6 (11.4) | 54.0 (8.1) | 54.1 (8.9) | 50.0 (12.2) | 54.7 (12.7) | 52.0 (13.8) | 52.1 (11.5) | 54.9 (11.1) | 57.5 (8.0) | 54.7 (9.7) | 54.7 (7.7) | 56.8 (13.8) | 51.9 (15.9) | 56.1 (9.9) | 48.5 (11.0) | 52.0 (7.8) | 51.7 (4.9) | 42.7 (14.6) | 50.4 (9.5) | 52.3 (10.3) | 43.3 (10.4) |
|  | extremely severe | 45.0 (12.0) | 45.9 (13.2) | 46.8 (8.7) | 44.7 (9.2) | 50.0 (13.2) | 41.6 (15.2) | 37.6 (10.7) | 43.9 (9.6) | 48.5 (7.4) | 47.2 (7.1) | 46.4 (5.9) | 46.8 (11.8) | 36.5 (7.8) | 34.9 (8.8) | 46.8 (15.2) | 40.3 (20.8) | 46.4 (10.4) | 42.5 (12.4) | 54.8 (14.8) | 52.5 (22.3)* | 52.3 (8.7)* |
| **GAD-7 category** | no | 73.6 (12.4) | 72.5 (13.3) | 72.4 (13.0) | 71.0 (13.1) | 75.1 (12.6) | 73.6 (12.0) | 75.4 (11.4) | 74.5 (11.5) | 73.1 (14.2) | 72.9 (12.4) | 72.6 (11.6) | 74.8 (11.6) | 75.7 (10.4) | 76.2 (10.4) | 72.8 (13.1) | 72.0 (12.6) | 71.5 (13.9) | 69.9 (13.9) | 75.3 (13.5) | 72.1 (12.8) | 74.8 (12.0) |
|  | mild | 63.8 (12.1) | 62.5 (13.1) | 63.6 (11.5) | 62.7 (12.7) | 64.4 (11.9) | 64.5 (12.9) | 65.8 (10.4) | 64.5 (11.9) | 64.4 (13.3) | 63.4 (11.4) | 63.8 (9.8) | 64.8 (12.0) | 63.6 (14.3) | 67.4 (10.6) | 62.8 (12.3) | 59.0 (12.3) | 63.9 (11.7) | 61.6 (14.9) | 63.8 (11.9) | 65.9 (10.3) | 62.0 (9.3) |
|  | moderate | 55.6 (12.0) | 54.2 (11.6) | 56.3 (8.3) | 54.9 (15.2) | 55.6 (11.5) | 55.0 (11.8) | 58.6 (13.2) | 58.0 (11.7) | 58.1 (12.3) | 58.0 (8.7) | 60.1 (11.4) | 55.1 (12.1) | 56.0 (12.8) | 63.2 (12.0) | 51.6 (11.5) | 50.2 (9.6) | 53.0 (6.7) | 47.9 (17.2) | 56.4 (10.8) | 52.5 (8.7) | 50.1 (11.3) |
|  | severe | 47.7 (12.1) | 46.5 (14.8) | 49.3 (9.8) | 50.5 (10.3) | 52.1 (12.3) | 41.1 (13.4) | 43.5 (13.0) | 47.2 (9.9) | 49.2 (8.1) | 48.8 (6.5) | 51.9 (8.1) | 48.9 (9.2) | 40.3 (10.9) | 40.0 (11.6) | 48.8 (16.2) | 42.8 (21.2) | 50.4 (14.6) | 46.5 (14.6) | 56.2 (15.5) | 44.0 (22.0)* | 57.7 (8.1)* |

Note: *means that responders in that subgroup were n<5; **denotes n=1 having no SD in the subgroup

**Journal:** The European Journal of Health Economics

**Title:** Population normative data for OxCAP-MH capability scores

**Authors:** Péter György Balázs¹, Agata Łaszewska², Judit Simon²³, Valentin Brodszky¹

1 – Corvinus University of Budapest, Department of Health Policy, Budapest, Hungary

2 – Medical University of Vienna, Department of Health Economics, Vienna, Austria

3 – University of Oxford, Department of Psychiatry, Oxford, United Kingdom

**Corresponding author:** Péter György Balázs – peter.balazs@uni-corvinus.hu
